# Exercise Interventions in Breast Cancer: Molecular Mechanisms, Physical Benefits, and Practical Recommendations

**DOI:** 10.3390/medicina61071167

**Published:** 2025-06-27

**Authors:** Vasiliki Michou, Stefanos Zervoudis, Panagiotis Eskitzis, Georgios Tsamos, Dimitra Vasdeki, Andriani Vouxinou, Anisa Markja, Georgios Iatrakis

**Affiliations:** 1 Department of Midwifery, School of Healthcare Sciences, University of Western Macedonia, Keptse, 502 00 Ptolemaida, Greece; vasilikimichou@yahoo.gr (V.M.); peskitzis@uowm.gr (P.E.); 2 Department of Midwifery, University of West Attica, Agioy Spyridonos 28, Egaleo, 122 43 Athens, Greece; giatrakis@uniwa.gr; 3 Department of Mastology, Rea Hospital, 175 64 Athens, Greece; anisamrk@gmail.com; 4 Division of Endocrinology and Metabolism and Diabetes Centre, First Department of Internal Medicine, Medical School, Aristotle University of Thessaloniki, AHEPA University Hospital, 546 36 Thessaloniki, Greece; tsamgeor@gmail.com; 5 Department of General Practice and Primary Health Care, Aristotle University of Thessaloniki, AHEPA University Hospital, Stilponos Kyriakides 1 St., 546 36 Thessaloniki, Greece; demivs14@gmail.com; 6 Minister of Education, 151 80 Athens, Greece; vouxinoy@gmail.com

**Keywords:** breast cancer, exercise, molecular mechanisms, functional capacity, quality of life

## Abstract

Exercise interventions are increasingly recognized as effective non-pharmacological strategies to improve clinical outcomes in patients with breast cancer. This review provides a comprehensive framework linking physical activity with breast cancer risk reduction, disease progression, and survivorship. We first outline the robust epidemiological evidence demonstrating that regular exercise significantly reduces breast cancer incidence, recurrence, and disease-specific mortality. The review then delves into the molecular mechanisms by which exercise exerts its protective effects, including modulation of sex hormones, metabolic hormones, systemic inflammation, oxidative stress, circulating microRNAs, and breast cancer-related DNA methylation. Furthermore, we summarize findings from clinical trials evaluating the effects of exercise on cardiorespiratory fitness, functional capacity, and quality of life in breast cancer patients. Emerging research on the synergistic potential of exercise with conventional cancer treatments and bioactive dietary components, particularly polyphenols such as saffron and curcumin, is also discussed. Finally, we present evidence-based exercise recommendations tailored to breast cancer patients, emphasizing the importance of individualized prescriptions to optimize safety and therapeutic benefit. Collectively, this review highlights the multifaceted role of exercise in breast cancer prevention, treatment, and survivorship.

## 1. Introducing the Framework Between Exercise and Breast Cancer

Breast cancer (BC) remains a significant global health challenge [1,2], being the most frequently diagnosed malignancy worldwide as of 2020 with 2.3 million new cases and 685,000 deaths annually, and global cancer prevalence is projected to rise by approximately 47% by 2040, further increasing the cancer burden. Among women, BC accounts for approximately 25% of all cancer diagnoses and 16.7% of cancer-related deaths, making it the leading cause of both incidence and mortality in most countries [3]. In the European Union (EU), around 380,000 new cases and 95,800 deaths were reported in 2022, representing 13.8% of all cancer cases and 16.7% of female cancer mortality [4]. Notable geographic variability in age-standardized incidence rates exists, with Belgium (194.0 per 100,000) and Denmark (171.2) reporting the highest rates [5].

Currently, BC management involves a combination of targeted therapies, such as hormonal treatment, radiation, chemotherapy, and surgery, tailored to disease type, stage, and progression [6]. Regardless of the type and combination of treatment, BC patients are confronted with various side effects such as fatigue, neuropathy, cardiotoxicity and reduced strength [7,8,9]. Physical side effects are often accompanied by psychological symptoms, such as depression and anxiety, which collectively impair the overall quality of life of BC patients [6,10]. Moreover, BC patients tend to reduce their physical activity levels during treatment and often remain physically inactive throughout the aftercare period [11,12]. However, the complexity and multifaceted nature of BC make it unlikely that a single complementary approach can effectively address both physiological and psychological challenges. Among these, exercise is particularly beneficial in preventing BC and maintaining physical fitness in patients and survivors [13,14,15,16,17,18,19,20]. Regular physical activity and structured exercise have demonstrated significant benefits in managing therapy-related side effects in BC patients, including reduced fatigue [16,21], improved cardiorespiratory fitness [22], and increased muscular strength [16]. Exercise not only mitigates the adverse effects associated with treatments such as chemotherapy and radiation but also enhances overall physical function and quality of life and lowers mortality rates. Lee et al. [23], by performing a meta-analysis of 24 studies, found that both pre- and post-diagnosis physical activity significantly reduces BC-specific mortality (BCM) and all-cause mortality (AM). High post-diagnosis physical activity was associated with 29% lower BCM and 39% lower AM, while high pre-diagnosis physical activity reduced these risks by 20% and 26%, respectively. Moderate levels of physical activity also showed protective effects. Notably, reduced physical activity after diagnosis was linked to the highest mortality risk, emphasizing the importance of maintaining or increasing physical activity following a BC diagnosis [23]. Furthermore, higher physical activity levels are consistently linked to a reduced risk of BC development and recurrence, as well as improved survival outcomes [24,25]. These positive effects are attributed to the modulation of metabolic pathways, reduction of systemic inflammation, enhancement of immune surveillance, and improved cardiovascular health, underscoring the critical role of exercise as an adjunctive therapeutic strategy in BC management [26].

Although few clinical and retrospective studies have investigated the effects of exercise on BC patients undergoing various treatment types and combinations, evidence suggests that increasing physical activity levels or maintaining moderate-to-high levels of physical activity over the long term may benefit both the physical and mental health of this vulnerable population. This review provides a comprehensive analysis of the molecular pathways through which exercise influences BC biology, focusing on mechanisms related to inflammation, oxidative stress, and metabolism. Interestingly, this review uniquely integrates exercise-induced exosomes with BC miRNA dysregulation, a nascent field with therapeutic potential. Additionally, it examines the impact of exercise on functional capacity, cardiorespiratory fitness, disease progression, and quality of life in BC survivors. Practical recommendations, clinical considerations, and the potential synergistic effects of combining exercise with specific BC treatments or dietary strategies are also discussed.

## 2. Exercise Reduces BC Risk and Recurrence and Enhances Survival

Exercise has been shown to play a variable role in reducing cancer risk across different cancer types, although no definitive causal relationship has yet been established. Nevertheless, numerous studies suggest that improvements in lifestyle, including physical activity, are associated with reduced cancer incidence [27]. Exercise training, which consists of repeated bouts of physical activity that disrupt whole-body homeostasis, induces systemic adaptations across various cells, tissues, and organ systems [28,29]. While much of the current research has focused on the biomechanical and metabolic adaptations in skeletal muscle, cardiovascular tissue, adipose tissue, and vasculature, relatively little is known about how exercise affects other tissues, including tumors [30]. Despite this knowledge gap, exercise is increasingly being proposed as a therapeutic strategy to enhance multiple outcomes in cancer patients. Although exercise intervention studies in oncology vary widely in design and population, the general consensus is that exercise yields improvements in both physiological parameters, such as cardiopulmonary fitness, body composition, and physical function, and in patient-reported outcomes like fatigue, sleep quality, and psychological well-being [31,32]. Importantly, emerging evidence suggests that exercise may also influence tumor biology directly, thereby potentially contributing to improved clinical outcomes in cancer care.

Numerous studies have demonstrated that regular physical activity is associated with a reduced risk of BC. A 2016 meta-analysis comprising 38 cohort studies concluded that higher levels of physical activity are significantly associated with a reduced risk of BC compared to lower levels of activity, indicating a robust inverse relationship between physical activity and BC incidence [33]. Spei et al. [13], in a more recent systematic review involving 25.563 BC survivors, found that those with the highest levels of physical activity had a 42% lower risk of all-cause mortality and a 40% lower risk of BC-specific mortality compared to those with the lowest activity levels, highlighting the significant survival benefits associated with post-diagnosis physical activity. Exercise interventions have been shown to significantly reduce BC risk in both premenopausal [34] and postmenopausal women [35]. Epidemiological studies consistently report an inverse association between physical activity and BC risk in postmenopausal women, with Howard et al. [36] indicating risk reductions ranging from 20% to 80%. Furthermore, evidence suggests that the protective effect of physical activity is more pronounced in women with postmenopausal hormone-sensitive BC compared to those with premenopausal hormone-insensitive subtypes [37,38].

Furthermore, the association between body mass index (BMI) and BC risk has received increasing attention in recent years, although the findings remain controversial mainly due to complex, potentially age- and menopause-dependent nature of the BMI–BC association. For instance, Liu et al. [39] in a dose–response meta-analysis of 12 prospective cohort studies involving over 22 million participants, found a weak but significant positive association between BMI and BC risk, with every 5 kg/m^2^ increase in BMI linked to a 2% increase in risk. However, subgroup analysis revealed that higher BMI may be protective for premenopausal women (SRR: 0.98). Guo et al. [40] observed that women with BMI ≥ 25 or ≥30 kg/m^2^ had significantly higher recurrence risks (RR: 1.09 and 1.15, respectively) compared to those with lower BMI. Overall, each 1 kg/m^2^ increase in BMI was associated with a 2% rise in recurrence risk. Similarly, Chen et al. [41] found that higher BMI was significantly associated with increased BC risk in postmenopausal women (RR: 0.94), with a 3% risk increase per 1 kg/m^2^ BMI increment. Geographical and ethnic factors may influence the relationship between BMI and BC, potentially due to variations in genetic susceptibility. There is a significant inverse association between BMI and BC risk among European premenopausal women, which aligns with the findings from the AICR/WCRF 2017 report [42]. Women with a normal BMI of 21.5 had a 2.3% lower risk of BC compared to those with higher BMIs. In contrast, among Asian women, a positive nonlinear association was observed when BMI exceeded 25 kg/m^2^, while no association was found among American women [41]. These results are of high importance as engaging in at least 3–5 h of exercise per week, particularly high-intensity activities, has been shown to significantly lower BC risk [38] and thus BMI. According to Cardio-Oncology Rehabilitation (CORe) programs, CORe interventions can significantly reduce BMI (*p* = 0.037), particularly in obese BC patients, with an average reduction of 3.1 kg/m^2^ (95% CI: 1.3–4.8) [43]. Additionally, CORe-related exercise programs combining aerobic and resistance training have also been shown to lower BMI in obese BC patients [44].

Physical activity has been consistently linked to improved outcomes in BC. A meta-analysis of 12.108 women with invasive BC found that pre-diagnosis physical activity reduced all-cause mortality by 18%, while post-diagnosis activity decreased BC mortality by 30% and all-cause mortality by 41% [45]. The benefits were especially notable in women with estrogen receptor (ER)-positive tumors, showing a 64% reduction in all-cause and 50% reduction in disease-specific mortality, with no significant effect in ER-negative cases [45]. Additional studies showed that more than 3 MET-hours/week of activity is associated with reduced recurrence and improved survival, with a dose–response trend [45,46,47,48,49]. In ER−/progesterone receptor (PR)− tumors, recurrence risk dropped by up to 50%, and all-cause and BC-specific mortality decreased by 67% and 49%, respectively. Holmes et al. [24] reported 5- and 10-year survival rates of 97% and 92% for women performing ≥9 MET-hours/week, compared to 93% and 86% in those below 3 MET-hours/week. These findings suggest that physical activity is a simple, cost-effective intervention with substantial potential to improve BC outcomes [45].

In addition, in BRCA1/2 mutation carriers, who face a lifetime BC risk ranging from 30% to 80%, physical activity appears to offer protective benefits, although the current evidence remains inconclusive. The variability in cancer penetrance among carriers suggests that modifiable lifestyle factors, such as regular exercise, may influence risk. This positions physical activity as a promising non-invasive strategy for supporting BC prevention in individuals with a high genetic risk [26]. For instance, Pollán et al. [50] explored the role of lifestyle and dietary factors as potential modifiers of BC risk in women carrying BRCA1 and BRCA2 mutations. Their findings, presented at the American Society of Clinical Oncology Annual Meeting, highlighted that modifiable behavioral factors, particularly those related to diet and lifestyle, may influence cancer risk even in genetically predisposed individuals. Although specific details of the associations were not fully disclosed in the abstract, the study underscores the growing body of evidence suggesting that adherence to healthy lifestyle practices, such as maintaining a balanced diet and engaging in regular physical activity, could potentially attenuate the elevated BC risk in BRCA1/2 mutation carriers. These insights support the rationale for integrating lifestyle counseling into risk-reduction strategies for this high-risk population. Table 1 summarizes findings from various studies involving BRCA1/2 mutation carriers.

## 3. Molecular Mechanisms Underlying Exercise in BC Patients

### 3.1. Sex Hormones and Exercise

#### 3.1.1. Estrogen and SHBG

The Endogenous Hormones and Breast Cancer Collaborative Group reported a significant increase in BC risk with higher levels of total, free, and non-sex-hormone-binding globulin (SHBG)-bound estradiol in postmenopausal women [60]. Over 97% of estradiol and testosterone circulate bound to SHBG and albumin, leaving only a small free fraction biologically active and capable of binding to steroid receptors. SHBG thus regulates sex steroid bioavailability and influences BC risk [61]. Estrogens promote cell proliferation and tumor development, partly through their metabolism into catechol-estrogen quinones (CE-Q) via cytochrome P450 enzymes. These reactive metabolites can form DNA adducts and free radicals, contributing to genotoxicity and carcinogenesis [62,63]. Estrogen also signals through membrane receptors to activate ERK pathways, promoting growth and inhibiting apoptosis. When bound to SHBG, estrogen may signal through the SHBG receptor to activate cyclic adenosine monophosphate (cAMP) and inhibit ERK, reducing harmful effects [64]. After menopause, ovarian estrogen production declines, but adipose tissue in obese women continues estrogen synthesis, potentially sustaining risk [65].

In premenopausal women, SHBG concentrations do not appear to be associated with BC risk [66]. In contrast, a significant inverse association has been observed between SHBG levels and BC risk in postmenopausal women [66]. One possible explanation, as suggested by Drummond et al. [67], is that SHBG may not play a major role in BC risk during the reproductive years. However, this dynamic shifts after menopause. In postmenopausal women, elevated estrogen levels have been linked to an increased risk of BC, while higher SHBG concentrations are associated with a lower risk. This may be due to SHBG’s role in reducing the bioavailability of circulating estrogens, thereby attenuating their mitogenic effects on BC in the absence of ovarian hormone regulation [67].

Physical activity has been consistently associated with reduced circulating estrogen levels, likely mediated by several mechanisms [68]. Several studies suggest that physical activity influences sex hormone levels in both premenopausal and postmenopausal women, potentially modifying BC risk. In premenopausal women, physical activity, especially when combined with low energy availability, may disrupt regular ovulatory cycles, thereby reducing endogenous estrogen exposure [69]. In a study of healthy premenopausal women, Emaus et al. [34] and Verkasalo et al. [70] found that higher levels of physical activity were associated with lower circulating estradiol (E2) levels; additionally, both physical activity and heart rate were linked to a metabolic risk score, which in turn was associated with daily E2 levels, suggesting key biological pathways connecting sedentary behavior to increased BC risk [34], while decreasing BMI was found to increase SHBG [70].

But how exactly does physical activity affect both SHBG levels and BMI? In general, physical inactivity tends to raise insulin levels, which can suppress hepatic SHBG synthesis, resulting in increased free sex hormones [71,72]. By improving insulin sensitivity and decreasing insulin resistance, both acute and chronic physical activity can restore SHBG production, thereby reducing the availability of bioactive estrogens and androgens [73,74]. A previous cross-sectional study that included 267 healthy postmenopausal women found that increased physical activity was associated with lower estrone, estradiol, and free estradiol levels, independent of adiposity. Notably, women with high BMI and low activity had the highest estrogen levels and lowest SHBG, while those with low BMI and high activity had lower estrogens and higher SHBG, emphasizing the combined effects of adiposity and exercise on hormone profiles. The authors concluded that these associations may, in part, explain the positive associations between obesity, higher BMI and sedentary lifestyle on BC risk [73]. These observations support the biological foundation of the early stages of the physical activity–sex hormone–BC pathway, suggesting that hormonal changes may underlie the protective impact of exercise on BC risk [67].

Based on the above studies, regular physical activity has been consistently shown to influence estrogen levels, a critical factor in BC development and progression. Evidence from randomized controlled trials indicates that higher volumes of aerobic exercise are associated with greater reductions in circulating estrogens. In both premenopausal and postmenopausal women, engaging in 150 to 300 min of aerobic exercise per week over several months leads to significant decreases in estradiol and estrone levels, with a clear dose–response effect observed—greater exercise duration results in more pronounced hormonal changes [37,75]. Additionally, interventions incorporating high-intensity interval training (HIIT) or moderate-to-vigorous continuous training, even over shorter durations ranging from 3 to 12 weeks, have been shown to improve body composition and metabolic health in young women, which may indirectly contribute to lower estrogen production through reductions in adipose tissue [76,77]. Collectively, these findings support the conclusion that both the intensity and duration of exercise play important roles in modulating estrogen levels, highlighting exercise as a viable strategy for BC risk reduction and hormonal regulation.

#### 3.1.2. Progesterone, Androgens and Cortisol

**Figure 1:**
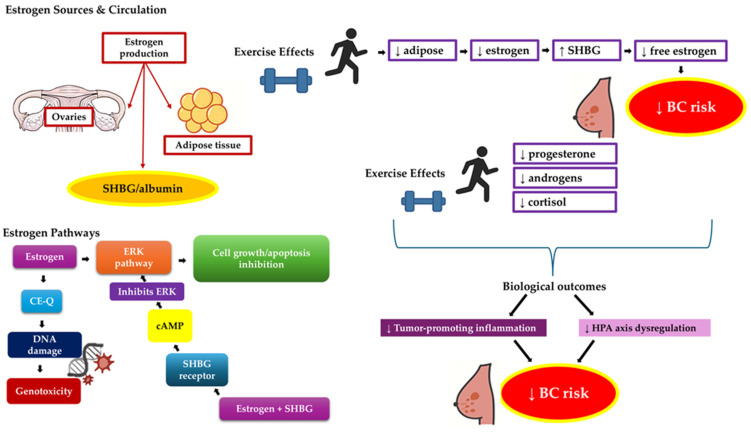
Molecular mechanisms of exercise modulate sex hormone pathways involved in breast cancer risk. ↑ = increase and ↓ = decrease. SHBG: sex hormone-binding globulin; CE-Q: catechol-estrogen quinones; cAMP: cyclic adenosine monophosphate; BC: breast cancer.

Physical activity may also modulate key hormones such as progesterone, androgens, and cortisol that are implicated in BC risk. Elevated progesterone exposure, particularly in postmenopausal women or during hormone therapy, has been associated with increased risk [78]. Exercise may reduce luteal-phase progesterone levels, potentially through alterations in ovulatory function, contributing to risk reduction [78]. Additionally, androgens such as testosterone and androstenedione have been linked to BC, especially in postmenopausal women, and higher physical activity levels are associated with reductions in these hormones [79]. In a meta-analysis by Swain et al. [78], acute exercise was found to variably affect cortisol levels: some studies reported no change and others observed increases (particularly with higher intensity and morning sessions), while some documented decreases post-exercise, more commonly in premenopausal women. Although cortisol may rise transiently with acute exercise, chronic training is associated with reduced baseline levels, indicating improved hypothalamic–pituitary–adrenal (HPA) axis regulation and a potential reduction in tumor-promoting inflammation [80,81,82]. Notably, no prospective studies have examined the long-term effects of physical activity on cortisol. These hormonal adaptations represent key mechanisms through which exercise may exert protective effects against BC [78,83]. Figure 1 illustrates the molecular mechanisms by which exercise modulates sex hormone pathways involved in BC risk.

### 3.2. Metabolic Hormones and Exercise

Elevated insulin, insulin-like growth factor 1 (IGF-1), and estrogen levels, alongside reduced IGF-binding proteins (IGFBPs) and SHBG, are associated with increased BC risk, recurrence, and mortality [84,85,86,87,88,89]. Physical activity, by lowering insulin and estrogen, may mitigate this risk [90]. To be more precise, physical activity exerts significant modulatory effects on metabolic hormones involved in carcinogenesis, including insulin, IGFs, and their binding proteins. These hormones play critical roles in cell proliferation, survival, and differentiation, and processes central to cancer development and progression [91]. In both BC and other cancer contexts, elevated levels of insulin and IGF-1 have been implicated in tumorigenesis via activation of mitogenic and anti-apoptotic signaling pathways, such as Ras-MAPK and PI3K-Akt [91,92,93]. Evidence from human and animal studies indicates that exercise-induced weight loss reduces circulating levels of IGF-1, insulin, and leptin, thereby downregulating IGF-1-related pathways and promoting cell cycle arrest and cancer suppression [92,93]. In postmenopausal women, aerobic exercise over six months has been shown to significantly lower IGF-1 and IGF-3 levels compared to non-exercising controls [86]. Furthermore, regular physical activity improves insulin sensitivity and lowers fasting insulin, glucose, total IGF-1, and HbA1c, while increasing levels of IGFBP-1 and IGFBP-3 [26,86,87,89,92,94]. These hormonal shifts are mediated not only by reductions in adiposity but also through direct effects of muscle contraction. Exercise enhances glucose uptake and insulin signaling via increased post-receptor signaling efficiency, elevated glucose transporter expression, augmented glycogen synthase and hexokinase activity, and improved muscle capillary density [89,92,95,96]. Importantly, these effects are observed even in the absence of significant body weight changes, underscoring the intrinsic value of muscle activity in modulating cancer-related metabolic pathways. Thus, physical activity represents a potent, non-pharmacological intervention to modify hormonal risk factors associated with BC and other malignancies.

### 3.3. Inflammation and Exercise

Regular physical activity induces anti-inflammatory effects by reducing visceral fat and promoting an anti-inflammatory environment. Myokines like Interleukin-6 (IL-6), released from contracting muscles, stimulate cortisol and adrenaline, which suppress pro-inflammatory cytokines. Additionally, exercise downregulates Toll-like receptors (TLRs) 1, 2, and 4, reducing inflammatory signaling [97]. Exercise also promotes a shift in macrophages from the pro-inflammatory M1 to the anti-inflammatory M2 phenotype, reducing TNF-α and IL-6 while increasing IL-10 and arginase. Animal studies, including that by Kawanishi et al. [98], indicate that exercise attenuates adipose tissue inflammation by reducing the infiltration of pro-inflammatory macrophages and CD8+ T lymphocytes. Additionally, chronic exercise has been shown to lower circulating levels of inflammatory monocytes and elevate regulatory T cells (Tregs), contributing to a systemic anti-inflammatory cytokine profile [98]. Human studies show lower CRP, IL-2, IL-6, and TNF-α and higher IL-10, IL-1RA, Tregs, and activated NK cells after exercise [26,27]. Collectively, these findings support the role of exercise in modulating immune function and mitigating inflammation relevant to cancer pathophysiology, including BC.

### 3.4. Oxidative Stress and Exercise

Still, regular exercise is recognized as a critical modifiable lifestyle intervention with therapeutic and preventive potential in individuals with BC, considering oxidative stress management and DNA damage. Karimi and Roshan [99] investigated the effects of 6 weeks of water-based exercise and ginger supplementation on oxidative stress and adiponectin levels in obese women with BC. Both interventions individually improved antioxidant markers [increased adiponectin, nitric oxide, glutathione peroxidase (GPx); reduced malondialdehyde (MDA)]. However, the combined intervention showed the most significant benefits. These findings support water-based exercise, especially when paired with ginger supplementation, as a potential non-drug strategy to reduce oxidative stress in this population. More recently, Delrieu et al. [100], by performing a 6-month personalized physical activity intervention in patients with metastatic BC, noticed an increase in GPx activity (+17%) and MDA levels (+9%). Physical activity and fitness improvements were positively associated with increased GPx and negatively with leukocyte NADPH oxidase (NOx). Patients with disease progression or death had higher MDA (+20%) and lower advanced oxidation protein products (AOPP, −46%). These findings suggest that regular exercise may enhance antioxidant defenses and reduce prooxidant activity, with MDA and AOPP as potential biomarkers of disease progression. Brown et al. [101], in a study that aimed to examine whether exercise and diet could reduce oxidative stress and prevent telomere shortening in BC survivors over 52 weeks, found that among 342 participants randomized to control, exercise, diet, or combined intervention, baseline telomere length was significantly shorter than age-adjusted norms, indicating accelerated aging. While exercise alone had no effect, both diet alone (−10.5%) and exercise plus diet (−9.8%) significantly reduced oxidative stress marker 8-iso-PGF2α. However, none of the interventions significantly affected telomere length. These findings suggest that dietary changes, with or without exercise, may reduce oxidative stress but do not impact telomere attrition in this population. The reason why the exercise may have null effects on telomere length may be the short exercise period, as telomere size seems to be longer in healthy endurance athletes compared to inactive ones [102], as well as the type of exercise (HIIT exercise seems to significantly increase telomere length in those who exercise) [103], non-healthy lifestyle (there are studies that link shorter telomere lengths to BC and energy excess) [104,105], the ongoing presence of oxidative stress itself and the lack of a targeted dietary protocol designed to optimize antioxidant defenses. Mechanistically, a diet high in pro-oxidant fats promotes lipid peroxidation, generating reactive oxygen species that preferentially damage guanine-rich telomeric DNA. Because telomeres have limited repair capacity, such oxidative lesions expedite telomere loss during cell division. Elevated biomarkers—particularly 8-iso-PGF_2_α—correlate with this increased oxidative burden and faster telomere shortening [106]. Together, these data imply a complex interplay between diet quality and exercise modality in modulating telomere dynamics, warranting longer and more precisely tailored interventions. Moreover, even though physical activity appears to enhance antioxidant levels in cancer patients, the low quality of existing evidence highlights the need for more robust, well-designed studies to confirm these findings.

### 3.5. Exercise-Induced Circulating microRNAs

Emerging evidence suggests that exercise training may influence circulating microRNA (c-miRNA) expression with potential anti-tumor effects in BC. Preclinical studies demonstrate that exercise reduces tumor incidence, size, and proliferation [107,108,109,110]. Some studies show that exercise-conditioned serum from animals or BC survivors reduces tumor cell viability in vitro [108,109]. Exercise may modulate c-miRNAs involved in tumor suppression. In mice, a 5-week exercise regimen combined with hormone therapy increased tumor-suppressive miRNAs (miR-206, let-7a) and decreased oncomiR miR-21, correlating with reduced tumor growth markers [111]. In humans, studies are limited. One study found changes in six c-miRNAs in BC survivors undergoing weight loss intervention including physical activity [112]. Another study showed increased miR-133a-3p in strength-training responders [113]. Alizadeh et al. [114] reported that exercise with hormone therapy decreased oncomiRs and increased tumor suppressor miRNAs, approaching levels in healthy controls. Likewise, Pulliero et al. [115] noticed that among 1900 miRNAs analyzed, 14 were found to be modulated by exercise, with the most notable changes being the upregulation of miR-206 and downregulation of anti-miR-30c—both of which are associated with breast cancer progression. Functional analysis in MCF-7 BC cells revealed that these miRNA changes inhibited cell growth and promoted apoptosis, particularly when combined, leading to cell cycle arrest at the G1/S phase. These findings suggest that exercise-induced modulation of specific miRNAs may contribute to its protective effects against BC and highlight the potential of circulating miRNAs as non-invasive biomarkers for cancer prevention [115]. However, no study has yet isolated the effects of exercise training alone on c-miRNA expression in BC patients, underscoring a gap for future research [116].

### 3.6. BC-Related DNA Methylation and Exercise

Bryan et al. [117] found that individuals who were more physically active and physically fit had lower levels of DNA methylation across 45 cancer-related CpG sites. Additionally, participants who increased their physical activity over 12 months showed reductions in DNA methylation. According to these, DNA methylation may be a biological mechanism through which exercise reduces cancer risk and could potentially serve as a biomarker in behavioral intervention studies. Given the fact that the nature and extent of the effect of physical activity on methylation of genes associated with BC risk remain an open question, Gillman et al. [118] investigated whether aerobic exercise influences DNA methylation of BC-related genes in a dose-dependent manner. Among 276 women, 137 completed a 16-week supervised aerobic intervention with varying intensity and duration, and 81 had viable methylation data. Total exercise volume was not linked to changes in methylation. However, greater improvements in cardiorespiratory fitness (VO_2_max) were associated with decreased methylation of BRCA1 (*p* = 0.01), and higher self-reported exercise during follow-up was linked to reduced GALNT9 methylation. Thus, enhanced physical fitness and consistent physical activity may impact DNA methylation patterns in genes associated with BC risk. Recently, Moulton et al. [119] found that a supervised online physical activity program in women undergoing treatment for primary BC modulated promoter-specific DNA methylation and gene expression related to antioxidant defense and cancer. Physical activity preserved superoxide dismutase (SOD) enzyme activity and significantly increased SOD2 mRNA expression (~77%), which was inversely correlated with decreased DNA methylation (~20%) at the SOD2 promoter. Similarly, physical activity reduced methylation at the L3MBTL1 promoter (~25%), leading to increased gene expression (~43%). This was accompanied by a 15% upregulation of TET1 mRNA and a 28% downregulation of DNMT3B, indicating shifts in the balance between methylation and demethylation. These epigenetic and transcriptional changes may enhance redox homeostasis, influence critical biological pathways, and potentially improve treatment response and quality of life in post-surgical BC patients.

**Figure 2:**
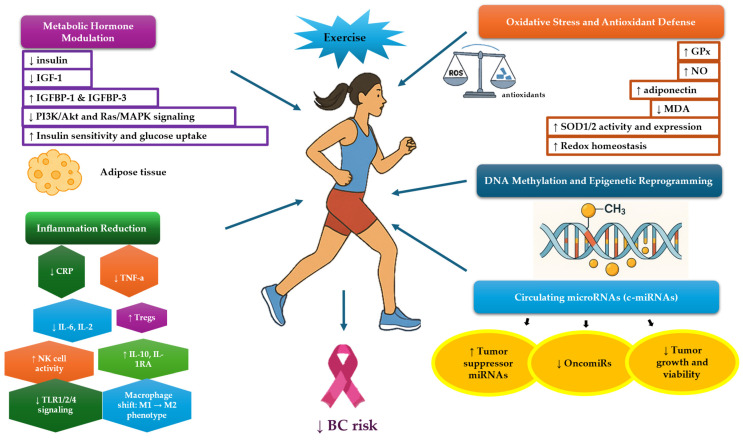
Molecular mechanisms of exercise in breast cancer. ↑ = increase and ↓ = decrease. IGF-1: insulin-like growth factor 1; IGFBP: IGF-binding protein; CRP: C-reactive protein; TNF-a: Tumor Necrosis Factor Alpha; NK: natural killer; GPx: glutathione peroxidase; ΝO: nitric oxide; MDA: malondialdehyde; SOD: superoxide dismutase; IL: Interleukin; TLR: Toll-like receptor.

Furthermore, the variability observed in BRCA1 and SOD2 methylation patterns in response to physical activity likely arises from complex interactions between tissue specificity, exercise timing, and intensity. Epigenetic responses to physical activity appear to be highly tissue-dependent. For instance, Gorski et al. [120] demonstrated that skeletal muscle from breast cancer survivors retains a hypermethylated profile even more than a decade post-treatment, distinct from tumor-specific methylation patterns. Notably, five months of aerobic training partially reversed these epigenetic changes in skeletal muscle, restoring DNA methylation at gene regulatory regions toward patterns observed in healthy, age-matched controls. This highlights the tissue-specific plasticity of the epigenome in response to exercise interventions [120]. Zeng et al. [121] reported consistent methylation patterns between breast tumors and blood, suggesting some cross-tissue similarity, after 6 months of moderate-intensity aerobic exercise and concluded that long-term exercise after a BC diagnosis may affect epigenetic regulation of tumor suppressor genes [121]. In contrast, Barres et al. [122] demonstrated that HIIT induces rapid DNA hypomethylation in skeletal muscle, suggesting that the timing and intensity of exercise critically influence the epigenetic response. Interestingly, the same exercise performed at a lower intensity (40% VO_2_ max) failed to elicit methylation changes, underscoring the unique potency of HIIT in driving epigenetic modifications. However, the impact of high-intensity interval training on BC-related DNA methylation remains largely unexplored, despite HIIT having been previously studied for its physiological and clinical effects in BC patients [123]. This represents a critical gap in understanding the epigenetic mechanisms underlying exercise-induced benefits in BC. Last but not least, cross-tissue comparisons have revealed strong correlations in DNA methylation patterns between blood, saliva, buccal tissues, brain, and breast [121,124,125]. However, studies specifically examining physical activity-induced methylation changes across these tissues remain limited. Figure 2 summarizes the main exercise-induced molecular mechanisms in BC.

### 3.7. Apoptosis/Angiogenesis and Exercise

Angiogenesis is a critical process for tumor growth, progression, and metastasis. Normally, the balance between pro- and anti-angiogenic factors regulates new blood vessel formation. In cancer, this balance shifts early toward a pro-angiogenic state, triggering an “angiogenic switch” that promotes new vasculature to support tumor expansion. Hypoxia-inducible factors (HIFs) play a key role by activating several pro-angiogenic factors, with vascular endothelial growth factor (VEGF) being central. VEGF not only directly stimulates blood vessel growth but also regulates other molecules involved in endothelial cell proliferation [126,127]. Additionally, several studies have demonstrated that hypoxia induces the upregulation of HIF-1α, which in turn increases the expression of VEGF, thereby promoting the formation of new blood vessels [128]. HIF-1 overexpression has been extensively reported in a broad range of solid malignancies, including ovarian, bladder, endometrial, breast, colorectal, glioma, pancreatic, renal, and prostate cancers [129]. HIF-1α plays a key role in cellular adaptation to the low-oxygen conditions of the tumor microenvironment. By activating genes involved in angiogenesis, invasion, metabolic reprogramming, and survival, HIF-1α contributes to tumor progression and metastasis. Multiple studies have shown that exercise reduces tumor hypoxia. In this context, the reduction in HIF-1α expression following exercise training may inhibit its pro-angiogenic activity. Isanejad et al. [111] found that interval exercise training combined with tamoxifen led to a greater reduction in HIF-1α and ER-α expression compared to the combination with letrozole. Furthermore, tamoxifen alone decreased the expression of both VEGF and HIF-1α. These findings suggest that exercise training may inhibit tumor angiogenesis primarily through modulation of the HIF-1α and ER-α signaling pathways [111]. In an orthotopic rat prostate cancer model, acute exercise doubled tumor blood flow and reduced hypoxic fraction by up to 15% [130,131]. Treadmill running increased microvessel density, improved vessel maturity, and lowered tumor hypoxia in breast cancer animal models compared to sedentary controls [132,133]. In mice with 4T1 tumors, chronic voluntary wheel running raised microvessel density by 50%, tripled pericyte coverage, and halved hypoxic regions [132]. These vascular changes were linked to calcineurin–NFAT–TSP-1 signaling activated by exercise-induced shear stress [130]. Although reduced hypoxia is expected to lower HIF-1α levels, some studies report that HIF-1α expression has increased following exercise despite improved oxygenation [133], suggesting alternative regulation mechanisms. Further research is needed to clarify these complex responses.

Ergun et al. [134] were among the first that investigated the effects of exercise on angiogenesis and apoptosis in BC patients. After 3-month exercise training, they found no significant differences between groups in any measured cytokine or growth factor levels. Most markers, including IL-6, TNF-α, VEGF, and thrombopoietin, did not significantly change from pre- to post-exercise in any group. Notably, IL-8 and epithelial neutrophil activating protein-78 significantly decreased only in the home exercise group. These changes in angiogenesis-related molecules suggested a potential effect of exercise on these pathways [134]. Exercise also appears to influence the growth of cancer cells, as serum from healthy individuals who exercise inhibits proliferation and activates apoptosis in various types of cancer cells [135]. Studies in BC patients, [129], have shown that exercise significantly reduced tumor growth in ER-negative and -positive BC mouse models. Their study revealed an increase in apoptosis by 1.4-fold, improved microvessel density and maturity, enhanced tumor perfusion, and lowered hypoxia [131]. Likewise, Banoon et al. [136] performed a study that investigated the impact of 12 weeks of aerobic exercise on tumor-related gene expression in BALB/c female mice with BC. After treadmill training, miR-15a expression increased 2.6-fold, while HIF-1α, B-cell lymphoma-2 (BCL-2), and VEGF expression significantly decreased by 3.1-, 2.6-, and 2.4-fold, respectively. Inhibition of BCL-2/VEGF could also be linked to clinical outcomes such as reduced breast tumor growth [132] and tumor cell apoptosis [137]. These findings indicate that physical exercise modulates critical molecular pathways regulating angiogenesis and apoptosis, thereby potentially attenuating the progression of BC [136].

### 3.8. Exercise-Induced Exosomes

Exercise’s anti-tumor effects are multifaceted but not yet fully understood [27,138]. Exercise induces the release of muscle-derived exosomes carrying over 300 molecules, including proteins, myokines, miRNAs, and glycolytic enzymes [139]. These exosomes can act as tumor suppressors by modifying cancer metabolism and other hallmarks of cancer [140,141]. Muscle-derived exosomes from exercise can directly interact with cancer cells, altering their structure and metabolic activity, and can also modulate the function of tumor-infiltrating immune cells to influence tumor growth [142]. Exercise-induced circulating molecules can counteract tumorigenesis by affecting the tumor microenvironment—comprising immune cells, mechanical and chemical stressors, and humoral factors, thus playing a key role in suppressing cancer progression [143]. Additionally, exercise-induced miRNAs interact with the Hippo Tumor Suppressor Pathway, which blocks oncogenic transcription factors Yes-Associated Protein (YAP) and Transcriptional Co-activator with PDZ-binding Motif (TAZ), reducing proliferation and survival of cancer cells [144,145]. In BC patients, the transcription of miR-223-3p is increased in BC cells. Its inhibition through exercise leads to reduced proliferation, migration, and invasion via the Hippo/YAP signaling pathway [146]. Additionally, catecholamines released during exercise activate the Hippo pathway, lowering BC risk [147].

Exercise also modulates key aspects of the BC microenvironment. Several studies have demonstrated that miR-486-5p, upregulated by exercise, plays a crucial role in reshaping the BC microenvironment. It enhances the immune response by promoting the recognition and targeting of tumor cells by cytotoxic T lymphocytes and natural killer cells in BC patients [148,149]. Moreover, emerging evidence suggests a promising role for exercise-induced miR-342-5p in BC progression and prognosis. Lindholm et al. [150] demonstrated that miR-342-5p inhibits HER2 signaling, and higher expression of this miRNA is linked to improved overall survival and extended time to recurrence in BC patients. Although the direct effects of exercise on circulating extracellular vesicle-packaged miRNAs (EVPs) in BC patients remain to be clarified, Hou et al. [151] observed significant increases in plasma exosomal miR-342-5p following a year of rowing training in humans and 4 weeks of swimming in rats. These findings raise the possibility that exercise-induced upregulation of miR-342-5p may synergize with other molecular pathways, such as MMP-2 activity modulation, to suppress HER2-driven tumorigenesis. Interestingly, while post-diagnosis exercise provides limited benefit for patients with ER-, progesterone receptor-, or HER2-negative breast tumors, the enhanced susceptibility of the HER2 pathway to miR-342-5p suggests that HER2-positive patients may particularly benefit from exercise interventions. However, this remains to be experimentally confirmed.

Recent evidence also supports the concept that exercise-released, myokine-packed extracellular vesicles (EVs) act as metabolic modulators within breast BC. In addition, we are now at the beginning of understanding the connections between muscle cell health, exercise, and cancer cell metabolism [152]. What we know is that muscle-derived EVs are rich in myokines, such as irisin, and cytokines can regulate the metabolism and proliferation of recipient cells [153]. Gannon et al. [154] found that irisin, an exercise-induced myokine, reduced BC cell proliferation, migration, and viability while sparing normal cells. Importantly, irisin enhanced doxorubicin-induced cytotoxicity and promoted apoptosis through caspase activation while concurrently suppressing NFκB signaling. Although the study focused on recombinant irisin, emerging evidence suggests that such myokines can be packaged within exosomes released during exercise, enabling endocrine-like signaling to distant tissues, including tumors. These findings suggest a potential mechanism by which exercise-derived, myokine-enriched exosomes, such as those containing irisin, may contribute to BC prevention and enhance chemotherapy responsiveness through apoptotic and anti-inflammatory pathways. Moreover, some studies suggest that these exercise-induced circulating myokines can directly suppress the growth of mammary cancer cells. Hojman et al. [109] found that serum collected from mice after physical exercise reduced proliferation by approximately 52% and increased apoptosis markers in MCF-7 BC cells. They identified oncostatin M (OSM) as a key myokine mediating these effects, with OSM treatment inhibiting proliferation by ~42% and boosting apoptosis by ~46%. In addition, in two triple-negative BC mouse models (4T1 and EO771), treatment with exercise-induced EVs derived from the plasma of healthy mice significantly delayed tumor progression, underscoring the potential of these vesicles in modulating tumor growth [155]. Collectively, little is known about how exercise-induced exosomes interact with BC cells and influence their metabolism, underscoring the need for further research on muscle-derived EVs and myokines in cancer progression, which could offer promising insights into the pathophysiology, progression, and therapeutic management of metastatic cancers. Table 2 provides an overview of exercise-induced biomarker alterations and their clinical implications for managing BC.

## 4. Exercise-Induced Effects in BC Patients: What Do We Know So Far?

### 4.1. Studies in BC Patients With or Without Related Lymphedema and Survivors

Several studies have been performed in both BC patients and survivors. In a recent systematic review, Ficarra et al. [156] observed that physical exercise interventions are increasingly recognized as effective in preventing the deterioration of cardiorespiratory fitness, muscular strength, fatigue, and health-related quality of life in individuals undergoing treatment for BC. In BC survivors, such interventions are also associated with significant improvements in these same outcomes. Among the different types of exercise, resistance training and combined exercise programs demonstrate the most promising effects on these key health indicators [156]. Lin et al. [157], by performing a systematic review and meta-analysis regarding exercise effects in BC patients, found that aerobic exercise significantly reduced pain intensity and improved shoulder flexion and internal rotation range of motion, upper limb function, and muscle strength (flexion and abduction) in postoperative BC patients. Shoulder–elbow movement exercises enhanced external shoulder rotation and significantly lowered the incidence of arm lymphedema. Additionally, resistance exercise contributed to improvements in upper limb function. These findings support the use of targeted exercise interventions to address common postoperative complications in BC survivors.

Exercise interventions have been shown to significantly benefit patients with BC-related lymphedema (BCRL), particularly in improving physical function and quality of life (QoL). Basha et al. [158] compared the effects of different exercise modes and found that both resistance and aerobic training improved shoulder range of motion, reduced pain intensity, and enhanced QoL, with the resistance group showing greater gains in muscle strength. Similarly, Park et al. [159] reported that progressive resistance exercise using a Thera-band significantly improved upper limb function and handgrip strength, with notable intragroup reductions in edema volume, although no significant intergroup differences were found. Improvements in global health status and role functioning were also more pronounced in the progressive resistance group compared to a home-based exercise group. Complementing these findings, Pasyar et al. [160] demonstrated that an 8-week yoga intervention led to significant improvements in physical, emotional, and role functioning, as well as reductions in fatigue, pain, and insomnia. However, no significant changes in limb volume were observed. Collectively, these studies support the efficacy of structured exercise, whether it be resistance training, aerobic activity, or yoga, in enhancing physical function and QoL in women with BCRL, while effects on edema volume remain variable across exercise modalities. However, several studies have reported conflicting findings regarding the effectiveness of various exercise protocols, largely due to common limitations such as small sample sizes (n < 100), heterogeneity in exercise interventions, lack of blinding, and low adherence to exercise sessions. Table 3 summarizes the findings from some studies that have been published within the last five years that have investigated exercise interventions in BC patients and survivors.

### 4.2. Studies in BRCA1/2 Carriers

The LIBRE study is a randomized controlled trial designed to evaluate whether lifestyle interventions can modulate cancer-related risk factors in women with germline BRCA1/2 mutations. As outlined by Kiechle et al. [168], the study protocol involves structured guidance aimed at improving adherence to a Mediterranean diet, increasing physical activity, and enhancing overall health metrics such as BMI and cardiorespiratory fitness. Building on this framework, Neirich et al. [169] investigated the potential modulatory effects of lifestyle factors on the RANK/osteoprotegerin (OPG) signaling axis in women carrying germline BRCA1/2 mutations. In this randomized controlled trial, 49 gBRCA1/2 mutation carriers were assigned to either an intervention group, which received structured guidance to improve physical fitness and dietary habits, or a control group receiving standard care. Over a 3-month period, serum levels of OPG significantly increased in both groups, while levels of soluble RANK ligand (sRANKL) showed a significant decrease only in the intervention group. Notably, increased omega-3 fatty acid intake was positively associated with OPG elevation, and baseline sRANKL levels were predictive of the magnitude of change during the intervention. These findings suggest that lifestyle modifications may influence key biomarkers involved in mammary carcinogenesis and could represent a non-pharmacologic strategy for cancer risk modulation in high-risk populations.

In a more recent study, Galasso et al. [170] examined the relationship between physical activity and sleep behavior in women carrying BRCA1/2 mutations, recognizing both as relevant lifestyle factors that may influence cancer risk and overall health in this high-risk population. In this observational study, participants’ physical activity and sleep patterns were assessed using actigraphy and validated questionnaires. The findings revealed that higher levels of physical activity were positively associated with improved sleep efficiency and longer total sleep duration. Conversely, sedentary behavior was linked to poorer sleep quality. These results suggest that promoting regular physical activity may not only contribute to physical health but also enhance sleep, which is increasingly recognized as a factor in cancer risk and survivorship. The study underscores the importance of a holistic lifestyle approach, incorporating both activity and restorative behaviors, in the management and potential risk-reduction strategies for BRCA1/2 mutation carriers.

## 5. Treatment or Dietary Strategies with Possible Synergetic Effects in BC

### 5.1. The Synergetic Effects of Treatment and Exercise Training

Jones et al. [171] developed the first animal model (triple-negative BC, MDA-MB-231 in nude mice) to assess exercise and BC treatment efficacy. Exercise alone improved 45-day survival (16%) compared to control (0%). However, combining exercise with doxorubicin reduced survival (20%) compared to doxorubicin alone (35%), indicating a potential antagonistic interaction [171]. Subsequent studies in female BC animal models demonstrated beneficial effects of exercise. Betof et al. [133] (ER−, 4T1 in immunocompetent mice) and Khori et al. [172] (ER+, MC4-L2 in immunocompetent mice) reported reduced tumor volume with exercise compared to control [~600 mm^3^ vs. ~800 mm^3^, *p* < 0.01 [133]; 0.37 cm^3^ vs. 1.43 cm^3^, *p* < 0.05 [172]]. Furthermore, combining exercise with treatment [cyclophosphamide [132]; tamoxifen [172]] enhanced therapeutic efficacy [~500 mm^3^ vs. ~600 mm^3^, *p* < 0.01 [133]; 0.25 cm^3^ vs. ~1.0 cm^3^, *p* < 0.05 [172]], indicating an additive effect.

Human studies have yielded inconsistent results. Courneya et al. [173] conducted the START trial, randomizing 242 BC patients (stage I–III) undergoing adjuvant chemotherapy to aerobic training (n = 78), resistance training (n = 82), or usual care (n = 82). During a median follow-up of 89 months, combining exercise groups showed a non-significant trend toward improved survival. The effect of exercise was more pronounced in HER2-positive patients and those completing ≥85% of chemotherapy. Overall, the exploration analysis of the START trial provides the first randomized evidence indicating that adjunctive exercise during chemotherapy may enhance BC outcomes [174]. In contrast, in a pilot trial, Rao et al. [174] evaluated supervised exercise during neoadjuvant chemotherapy (NC) for BC. Exercise reduced BMI compared to NC alone (28.0 vs. 35.8, *p* = 0.03) and showed a non-significant trend toward lower Ki-67 levels (7% vs. 29%, *p* = 0.14), indicating feasibility and potential survival benefit. Tumor size and C-peptide levels were comparable between groups. Likewise, Kirkham et al. [175], in a single-arm intervention with 73 stage I–IIIA BC patients undergoing supervised aerobic and resistance training compared to controls (n = 85), found that after a median follow-up of 70 months, disease progression (11% in both groups, *p* = 0.97) and overall mortality (7% in both groups, *p* = 0.78) were similar.

### 5.2. The Synergetic Effects of Polyphenol and Exercise Training in BC Patients [176]

#### 5.2.1. Saffron and Exercise Training

SOD is a key enzyme in the human antioxidant defense system and a potential therapeutic target for ROS-mediated diseases, including cancer. Saffron-derived carotenoids, crocin (Cro) and crocetin (Crt), exhibit antioxidant and anti-tumor effects, demonstrating significant radical scavenging capabilities. In MCF-7 BC cells, Cro and Crt inhibit SOD activity, with Cro neutralizing superoxide radicals and Crt interacting with the enzyme’s copper-binding site. However, in breast tumors of BALB/c mice, Cro and Crt enhance SOD activity after one month of treatment, potentially compensating for reduced SOD activity typically observed in cancer cells [177].

Crocin also inhibits nuclear factor kappa-light-chain-enhancer of activated b cells (NF-κB) activation, reducing cell viability, proliferation, and inflammation in BC cells by downregulating PRKCQ expression. Activation of the PRKCQ-dependent NF-κB pathway counteracts Cro’s effects, suggesting that Cro suppresses proliferation and inflammation via NF-κB inhibition [178]. In triple-negative metastatic BC cells (4T1), Cro and Crt reduce cell viability, invasion, migration, and adhesion, with Cro inhibiting migration more effectively and Crt showing stronger antiadhesion effects. Crocin’s antimetastatic properties are linked to modulation of the Wnt/β-catenin signaling pathway [179]. In the MDA-MB-231 cell line, crocin decreases the expression of β-catenin, Snail, Vimentin, and Zeb-1 while increasing E-cadherin, highlighting its role in inhibiting migration through Wnt/β-catenin pathway modulation [180].

**Figure 3:**
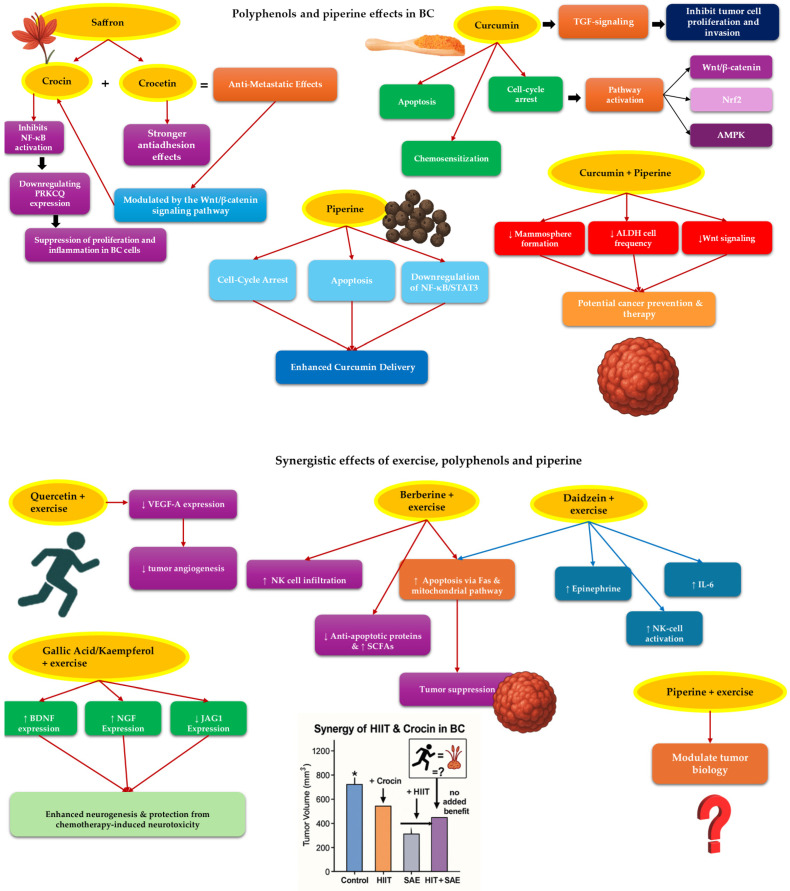
Individual and synergistic effects of polyphenols, piperine, and exercise on cancer. Note: Focus on molecular targets and clinical endpoints reviewed elsewhere. ↑ = increase and ↓ = decrease. * Statistically significant. BC: breast cancer; BDNF: brain-derived neurotrophic factor; NGF: nerve growth factor; IL-6: Interleukin 6; HIIT: high-intensity interval training; Nrf2: Nuclear Factor Erythroid 2-Related Factor 2; SAE: saffron aqueous extract; NK Cell: natural killer cell; SCFAs: short-chain fatty acids; NF-κB: nuclear factor kappa-light-chain-enhancer of activated B cells; AMPK: AMP-Activated Protein Kinase.

Regarding the synergetic effects of saffron consumption and exercise training, animal studies have demonstrated that HIIT and saffron aqueous extract (SAE) independently exhibit beneficial effects on BC outcomes in mice. Nezamdoost et al. [181] found that four weeks of HIIT increased SIRT1 and p53 mRNA levels in BC tissues, suggesting potential tumor suppression through p53 overexpression. However, combining HIIT with SAE did not enhance these effects, indicating that the combination may act through different pathways [181]. Higher doses of SAE supplementation and longer periods of HIIT or its combination with other exercise types may yield more promising results in cancer treatment. While Ahmadabadi et al. [182] observed that in cancer cachexia models, HIIT and SAE individually reduced weight loss, decreased apoptotic markers (caspase-3, Bax), and increased anti-apoptotic Bcl-2 levels. However, the combined intervention did not mitigate muscle wasting or apoptosis, suggesting that either treatment alone may be more beneficial for cancer-related muscle loss [182]. Additionally, tumor volume reduction was observed with HIIT and SAE individually, but the combination did not significantly increase apoptotic markers compared to single treatments, indicating limited synergistic effects [183] (Figure 3).

In human studies, evidence is limited, but a clinical trial by Mirzaei et al. [184] evaluated saffron’s impact on cancer-related fatigue in BC patients. Saffron-containing Jollab significantly improved fatigue scores after four weeks compared to placebo, as measured by the Visual Analog Fatigue Scale (VAFS) and Fatigue Severity Scale (FSS). Physical and cognitive fatigue levels were notably reduced, while the affective component remained unaffected. These findings suggest that saffron supplementation may be beneficial for managing fatigue in BC patients.

#### 5.2.2. Curcumin and Exercise Training

Curcumin exhibits significant anti-proliferative effects in BC models by inhibiting the growth of hormone-dependent, hormone-independent, and multidrug-resistant (MDR) cell lines through G2/S-phase cell cycle arrest. It also reverses adriamycin resistance in MCF-7 cells, suggesting potential in overcoming chemoresistance [185]. Additionally, in HER-2/neu-overexpressing SK-BR-3 cells, curcumin induces G2/M-phase arrest, upregulates p21, downregulates cyclin D1, and triggers apoptosis within 24 h [186]. Furthermore, curcumin modulates multiple molecular signaling pathways, including Wnt/β-catenin, Nrf2, AMPK, and MAPK, and shows therapeutic potential by regulating TGF-β signaling, which may inhibit tumor cell proliferation and invasion [187]. Furthermore, curcumin has been shown to alter noncoding RNA (ncRNA) expression, including long noncoding RNAs (lncRNAs) and circular RNAs (circRNAs), which regulate gene expression at the post-transcriptional level across various cancers [188].

In animal models, combining curcumin with exercise significantly inhibited BC growth compared to either intervention alone. This synergy was linked to modulation of key signaling pathways, including IL-17, PI3K-Akt, Wnt, calcium signaling, and metabolic pathways related to amino sugar metabolism [189,190,191]. Additionally, this combination reduced inflammatory markers (NF-κB, TNF-α), enhanced anti-tumor immune responses, and improved angiogenesis regulation via miR-126 and Angiopoietin-1 modulation [189,190]. While some studies reported no significant effect on oxidative stress markers, others found that curcumin and exercise decreased apoptosis in liver tissue and reduced cardiac apoptosis induced by doxorubicin through regulation of CAS3, CAS9, BAX, and BCL2 gene expression [190,191,192,193,194] (Figure 3).

In human studies, the combination of curcumin and aerobic exercise was found to reduce inflammation markers (hs-CRP, PTX3), decrease body fat percentage and BMI, and enhance quality of life in overweight BC survivors post-radiotherapy [195]. Additionally, curcumin supplementation demonstrated protective effects against tamoxifen-induced non-alcoholic fatty liver disease in ER+ BC patients, suggesting a potential preventive role in combination with conventional therapy [196]. While preclinical studies indicate promising synergistic effects of curcumin and exercise in BC management, clinical evidence remains limited, highlighting the need for more human trials to validate these findings. Future studies should implement randomized controlled trials with triple-negative BC to test whether adding daily curcumin to a long-term HIIT or endurance program yields greater reductions in the proportion of tumor cells that are actively dividing (as measured by the Ki-67 proliferation index) than HIIT alone.

#### 5.2.3. Other Substances and Exercise Training

Research on the combined effects of quercetin and exercise has shown that this combination can significantly reduce tumor angiogenesis by lowering VEGF-A expression in BC mice models. This indicates that quercetin, when paired with aerobic exercise, has an inhibitory effect on tumor growth. In contrast, exercise alone demonstrated only a limited impact [197,198]. Berberine combined with exercise seems to enhance immune function by increasing NK cell infiltration and promoting apoptosis through both Fas death receptor and mitochondrial pathways. It also reduce anti-apoptotic proteins and increased short-chain fatty acids (SCFAs), indicating potential tumor suppression [199,200]. Daidzein and exercise together inhibited tumor growth more effectively than alone, likely by increasing epinephrine, IL-6, and NK cell activation, which triggered apoptosis via the Fas/FasL pathway [201]. Gallic acid and kaempferol with exercise improve neurogenesis affected by chemotherapy, enhancing brain-derived neurotrophic factor (BDNF) and nerve growth factor (NGF) expression while reducing JAG1, indicating protection against neurotoxicity [202] (Figure 3).

Currently, there is limited clinical evidence regarding the effects of combining quercetin, berberine, daidzein, gallic acid, or kaempferol with exercise in BC patients. Most of the available data comes from animal studies, which suggest potential anticancer benefits, but their applicability to humans remains uncertain. While these compounds have shown promising effects, such as reducing tumor growth, enhancing immune response, and improving neurogenesis, no human clinical trials have confirmed their efficacy in BC treatment or supportive care. Future research should focus on conducting robust clinical trials to establish safety, optimal dosing, and therapeutic potential.

#### 5.2.4. What Is More to Be Explored?

However, studies have also shown the favorable effects of *Phellinus linteus* (PL). PL has demonstrated potential anticancer effects in BC models. Studies have shown that PL synergizes with chemotherapy agents like 5-fluorouracil (5-FU) to inhibit BC cell growth by inducing autophagy-related cell death without triggering apoptosis [203]. PL also suppresses tumor proliferation, angiogenesis, and invasiveness by causing S-phase cell cycle arrest and inhibiting the AKT signaling pathway, including downregulation of uPA and VEGF [204]. Additionally, combining PL with natural compounds, such as extracts from Smilax corbularia, enhances antioxidant and antiproliferative effects, indicating potential combinatorial benefits [205]. While these findings highlight the therapeutic potential of PL in BC treatment, the effects of combining PL with exercise remain unexplored. Given that exercise has been shown to modulate tumor biology and enhance treatment efficacy in various cancer models, future research should investigate the potential synergistic effects of PL and physical activity in BC management.

Piperine (PIP) has also been found to play a significant role in cancer. Piperine exerts potent anti-breast-cancer activity across both triple-negative (4T1, MDA-MB-231, MDA-MB-468) and ER-positive (MCF-7, MCF-7-10A, SKBR3, T-47D) cell lines by engaging multiple complementary mechanisms. It induces cell cycle arrest and triggers apoptosis, thereby halting proliferative capacity, while simultaneously modulating key signaling proteins (such as kinases and survival mediators) and downregulating oncogenic transcription factors like NF-κB and STAT3 [206,207,208,209]. Piperine enhances curcumin’s anti-tumor efficacy by improving its bioavailability, inhibiting metabolic clearance, and facilitating more effective tumor-targeted delivery [210]. This combination also lowers plasma levels of IL-10, and miR-21, although the precise molecular interactions underlying these effects remain to be elucidated [211,212]. In BC, combined curcumin and piperine dose-dependently halved mammosphere formation, serial passaging, ALDH^+^ cell frequency, and Wnt signaling at 5 µM—and abolished them at 10 µM—without affecting differentiation, underscoring their joint potential as cancer-preventive agents [213]. Despite compelling evidence that piperine can synergize with exercise to modulate tumor biology, no clinical trials have yet explored this combination in cancer patients. However, exercise physiology studies in healthy subjects demonstrate that peri-exercise supplementation with curcumin and piperine attenuates certain markers of muscle damage, although it does not completely abrogate all indices of exercise-induced tissue injury [214] (Figure 3).

Additionally, polyphenols such as catechins, anthocyanins, and proanthocyanidins act as prebiotics in animals by increasing beneficial gut microbes (i.e., Lactobacillus, Bifidobacterium), alongside increased production of SCFAs such as butyrate. According to Alves-Santos et al. [215], butyrate has a dual role in the gut–tumor axis. On the other hand, butyrate produced through microbial fermentation of dietary polyphenols enhances the bioavailability of these compounds by promoting gut barrier integrity and modulating microbial enzymatic activity. On the other hand, butyrate exerts potent anti-inflammatory effects by inhibiting histone deacetylases (HDACs) and reducing NF-κB activation, thereby attenuating systemic and local inflammation. These actions may contribute to both cancer prevention and treatment efficacy. Although preclinical studies provide strong evidence for the prebiotic and immunomodulatory effects of polyphenol–butyrate interactions, clinical evidence in humans remains limited. Clinical trials have also demonstrated comparable changes in gut microbiota composition, a decrease in inflammatory markers [215], and an increase in butyrate following polyphenol supplementation [216,217]. These results are of high importance, since butyrate plays a significant and multifaceted role in human health regulation [218]. To be more precise, extensive research has focused on the anti-tumor properties of butyrate [219]. Functioning as a tumor suppressor, butyrate exerts its effects by modulating immune responses and influencing the behavior of tumor cells as well as healthy intestinal epithelial cells [218]. Polyphenol metabolites produced by the microbiome have greater bioavailability and anticancer potency in breast tissue, indicating that butyrate-mediated enhancement of polyphenol absorption could improve outcomes in BC prevention or therapy [220]. Additionally, studies have shown that exercise induces a unique shift in the gut microbiota that is different from dietary effects [221] and increases butyrate production [222,223]. Although the individual effects of exercise, butyrate production, and polyphenol bioavailability have been studied separately, there has been no research to date that directly examines their combined impact on BC prevention and treatment. Nonetheless, the mechanistic framework known as the exercise–microbiome–butyrate–polyphenol bioactivation axis appears to be a promising target for future intervention studies aimed at preventing and treating BC.

## 6. Exercise Recommendations for BC Patients

Exercise training is increasingly recognized as a critical component of supportive care in cancer, offering a spectrum of benefits that extend well beyond general health improvements. While the role of exercise in managing chronic diseases such as cardiovascular disease and type 2 diabetes is well established, mounting evidence supports its direct, cancer-specific effects in patients. These include modulation of tumor biology, reduction of systemic inflammation, improvement in immune surveillance, and enhancement of treatment tolerance and efficacy. Importantly, moderate- to high-intensity endurance training has been shown to activate sympathetic pathways and mobilize cytotoxic immune cells, mechanisms that may suppress tumor growth and metastasis. In parallel, resistance training may counteract cancer-associated muscle waste (cachexia), supporting musculoskeletal integrity during and after treatment. For patients with cancer, exercise has been shown to improve physical function, reduce fatigue, enhance quality of life, and lower the risk of recurrence. Despite the compelling evidence and the safety of exercise interventions across all stages of disease, implementation in routine oncology care remains limited, often due to organizational and systemic barriers rather than scientific uncertainty. To realize the full potential of exercise in cancer care, it must be prescribed in a personalized and structured manner, aligned with the patient’s functional capacity and clinical status, and delivered by trained professionals within multidisciplinary settings. As the molecular mechanisms underlying exercise-induced anticancer effects become clearer, future guidelines will better define the optimal dose, intensity, and modality of exercise, reinforcing its role not merely as supportive care but as an essential adjunct to conventional cancer therapies [110].

The American College of Sports Medicine recommends moderate aerobic training (30 min, three times per week) and resistance training (8–15 reps at 60% of 1-RM, twice weekly) for cancer survivors [224]. The Exercise and Sports Science Australia (ESSA) position underscores the importance of exercise as a vital component in the holistic management of cancer patients, advocating for its integration into standard care protocols through a moderate- to high-intensity, multimodal, and individualized approach tailored to therapy cycles and patient characteristics [225]. Moreover, higher levels of post-diagnosis physical activity are associated with a lower risk of BC-specific mortality in patients with ER+/PR+, ER−/PR−, and triple-negative BC subtypes [226]. Thus, these patients are advised to perform 150 to 300 min of moderate-intensity aerobic activity or 75 to 100 min of vigorous aerobic activity each week, or a combination of both intensities, to lower estrogen, according to the U.S. Department of Health and Human Services Physical Activity Guidelines for Americans, 2nd edition [227]. However, despite these guidelines, about two-thirds of patients fail to meet the recommended activity levels [49]. The Exercise and Sports Science Australia (ESSA) position statement, as detailed by Hayes et al. [225], provides comprehensive guidelines on integrating exercise into cancer management, including for BC patients. It emphasizes that exercise should be considered a core component of standard cancer care due to its multifaceted benefits.

### Key-Recommendations

- Individualized Exercise Prescription: Exercise programs should be tailored to each patient’s specific cancer type, stage, treatment regimen, and overall health status. This personalization ensures safety and maximizes therapeutic benefits.
- Multimodal Approach: A combination of aerobic and resistance training is advocated. Moderate- to high-intensity exercises are generally appropriate, but the exact regimen should be adjusted based on individual capabilities and treatment phases.
- Safety and Feasibility: Prior to initiating an exercise program, a thorough assessment should be conducted to identify any contraindications or limitations. This ensures that the prescribed exercises are both safe and feasible for the patient.
- Integration Across the Cancer Care Continuum: Exercise should be incorporated at all stages of cancer care—pre-treatment, during treatment, and post-treatment, to mitigate side effects, enhance physical function, and improve quality of life.
- Addressing Barriers: Recognizing and addressing potential barriers to exercise, such as fatigue, psychological distress, or logistical challenges, is crucial. Providing behavioral support and education can enhance adherence and outcomes.

## 7. Conclusions

In conclusion, our review unveiled that exercise significantly reduces BC risk and recurrence and improves survival by modulating several key molecular mechanisms. It lowers bioavailable estrogens through increased SHBG, improves insulin sensitivity, decreases pro-inflammatory cytokines, and enhances antioxidant defenses, collectively creating a less favorable environment for tumor development and progression. Emerging evidence also suggests that exercise influences circulating microRNAs and DNA methylation patterns related to BC, highlighting its complex role in cancer biology. Notably, it is the first review to discuss the link between exercise-induced exosomes and BC miRNA deregulation, even though other recent studies have already explored the theme of exosomes and miRNAs as mediators of exercise in cancer patients. Furthermore, combining exercise with polyphenols, such as curcumin and saffron, may produce synergistic anticancer effects; however, further research is necessary to validate these findings. Several exercise-correlated societies in sports science and medicine have given practical exercise guidelines for BC survivors. Despite these clear guidelines, many patients fail to meet the recommended activity levels, underscoring the need for more effective implementation strategies. However, this comprehensive review acknowledges some limitations based on the research data. Many studies emphasize clinical outcomes over detailed molecular mechanisms, leaving gaps in understanding exercise’s precise effects on BC biology. Limited diversity in patient characteristics and potential publication bias restrict generalizability and long-term insights. Confounding factors like diet and comorbidities may affect results, and current recommendations are based on limited high-quality evidence, highlighting the need for rigorous randomized trials to refine exercise guidelines for BC patients. To enhance our understanding of the role of exercise in BC biology, future studies should prioritize a comprehensive investigation of the exercise-induced molecular mechanisms underlying clinical outcomes in BC patients. Research should encompass a more diverse patient population to ensure findings are generalizable across different demographics. Future studies should systematically vary exercise modality, intensity, and frequency to pinpoint regimens that not only enhance treatment efficacy but also synergize with dietary interventions, such as piperine and curcumin supplementation or Pl extracts, to maximize anti-tumor benefits. Lastly, mechanistic studies on exercise-induced reactive oxygen species (ROS) and their impact on PARP inhibitor (PARPi) sensitivity in BRCA-mutated BC models are also of high importance, as they may reveal novel synergistic interactions and inform personalized therapeutic strategies.

## Figures and Tables

**Table 1:** Studies examining the relationship between physical activity and BC risk in BRCA1/2 mutation carriers.

Authors, Year	Design	Sample Size	Assessment of Physical Activity	Main Results
Kehm et al. [51], 2020	Prospective cohort study	N = 15.550 women (6.503 families); 659 BRCA1, 526 BRCA2 carriers; 59% premenopausal	Physical activity questionnaire; METs estimation (hours/week).	Recreational physical activity in adulthood (highest 4 quintiles vs. lowest) associated with 20% lower BC risk (HR = 0.80). No association for adolescent activity. Effects not modified by familial risk or BRCA mutation status.
Lammert et al. [52], 2018	Case–control study	N = 443 matched pairs of BRCA mutation carriers	Nurses’ Health Study II Physical Activity Questionnaire; METs estimation (hours/week): overall (ages 12–34), adolescence (ages 12–17), early adulthood (ages 18–34).	No association between total physical activity and BC risk overall. Moderate physical activity at ages 12–17 associated with 38% lower risk of premenopausal BC (OR = 0.62). No association with postmenopausal BC.
Grill et al. [53], 2017	Randomized, prospective study	N = 68 mutation carriers; BRCA1 (61.8%) and BRCA2 (32.2%)	Interviews and questionnaires: type of physical activity, hours per week, and participation in sports.	Higher adolescent physical activity linked to lower cancer prevalence (*p* = 0.019); smoking status associated with cancer (*p* < 0.001); diseased carriers had lower BMI (*p* = 0.079) and lower physical activity than non-diseased carriers (*p* = 0.046).
Pijpe et al. [54], 2010	Nationwide retrospective cohort study	Ν = 725, including 218 diagnosed with BC within the past 10 years	Lifetime sport type, weekly hours, age at practice (≥6 months, ≥1 h/wk.); METs assigned per Ainsworth et al. [55] for intensity analysis.	Sports activity linked to reduced BC risk in BRCA1/2 carriers. Medium lifetime intensity (11–22.7 MET-h/week) showed significant risk reduction (HR = 0.59). Strongest inverse association seen for activity before age 30 (HR = 0.58–0.60). No clear dose–response relationship; post-30 activity also protective (HR = 0.63).
Friedenreich et al. [56], 2009	Prospective cohort study	Ν = 1.231 BC cases (1995–1997) followed ≥8.3 yrs for progression, recurrence, and new primaries	In-person baseline interviews; METs assigned per Ainsworth et al. [57] for intensity analysis.	Average total physical activity: 126 MET-h/week. Higher recreational physical activity was associated with lower BC death risk (HR = 0.54), especially at moderate (0.56) and vigorous intensity (0.74). Moderate-intensity recreational activity also reduced the risk of recurrence, progression, or new primary cancer (0.66). No association was observed between other physical activity types and BC survival. Recreational activity before diagnosis was particularly protective.
Nkondjock et al. [58], 2006	Case–control study	N = 80 French-Canadian families (250 members): 89 BRCA carriers with BC, 48 unaffected carriers	Interviews for physical activity assessment; METs assigned per Ainsworth et al. [57] for intensity analysis. FFQ to ascertain dietary intake.	Higher total energy intake linked to increased BRCA-related BC risk (OR = 2.76); no link found for other nutrients. Greater weight gain since age 18 (OR = 4.64) and 30 (OR = 4.11) and older age at max BMI (OR = 2.90) were also associated with higher risk. No association with BMI, smoking, or physical activity.
King et al. [59], 2003	Cross-sectional study	N = 1008 index cases	Questionnaire: categorized as active vs. inactive during adolescence and young adulthood.	Lifetime BC risk among female mutation carriers was 82%; risk by age 50 increased from 24% (born < 1940) to 67% (born ≥ 1940). Ovarian cancer risk: 54% (BRCA1), 23% (BRCA2). Adolescent exercise and low obesity are linked to delayed onset.

Note: BC: breast cancer; HR: hazard ratio; ΜΕΤs: Metabolic Equivalent of Tasks; FFQ: Food Frequency Questionnaire; OR: Odds Ratio; ΒΜΙ: body mass index.

**Table 2:** Exercise-induced biomarker responses and their clinical relevance in breast cancer management.

Biomarker	Exercise Response	Clinical Utility
CRP	↓ with aerobic	Monitor systemic inflammation
miR-133a	↑ with resistance	Predict muscle strength adaptations
Estrogen	↓ with aerobic (dose–response)	Modulate BC risk Target for prevention
SHBG	↑ with aerobic (via improved insulin sensitivity)	Reduces bioavailable estrogens Protective role in menopause
Testosterone/Androgens	↓ with aerobic and resistance training	Reduce pro-cancer signaling
Cortisol	Varied response ↓ with chronic exercise	Modulates HPA axis and inflammation
Insulin	↓ with aerobic and resistance training (weight loss required)	Reduce mitogenic signaling and insulin resistance
IGF-1	↓ with aerobic and resistance training (reduced cancer signaling)	Downregulate growth-promoting pathways
miR-486-5p	↑ with exercise	Enhances anti-tumor immunity Potential non-invasive market for BC prognosis
miR-342-5p	↑ with exercise (miR-342-5p targets HER2, modulates tumor microenvironment)	Potential target for HER2 + BC therapy
Irisin	↑ with exercise	Reduces BC cell viability, promotes apoptosis Potential therapeutic and chemosensitizer in BC
VEGF	↓ with aerobic and interval training	Modulates angiogenesis Potential marker for tumor vascular response
SOD-2	↑ expression, ↓ promoter methylation with aerobic training	Enhances antioxidant defense Potential biomarker for oxidative stress response in BC
HIF-1α	↓ with aerobic and interval training	Inhibits angiogenesis Marker of tumor hypoxia modulation
BCL-2	↓ with aerobic exercise	Pro-apoptotic shift Potential marker of exercise-induced tumor suppression
miR-206	↑ with combined exercise and hormone therapy	Tumor suppression miRNA Involved in proliferation and apoptosis regulation Anti-angiogenesis effects
let-7a	↑ with exercise and hormone therapy	Tumor suppression miRNA Regulates oncogenes and inhibits cancer cell growth Anti-angiogenesis effects
miR-21 (oncomiR)	↓ with exercise and hormone therapy	Ongogenic miRNA Reduction linked to decreased proliferation and metastasis

Note: ↑ = increase and ↓ = decrease. CRP: C-reactive protein; miR/miRNA: microRNA; BC: breast cancer; SHBG: sex hormone-binding globulin; HPA axis: hypothalamic–pituitary–adrenal axis; IGF-1: insulin-like growth factor 1; VEGF: vascular endothelial growth factor; SOD-2: superoxide dismutase 2; HIF-1α: hypoxia-inducible factor 1-alpha.

**Table 3:** Studies that have investigated exercise interventions in BC patients and survivors.

Authors, Year	Design	Sample Size	Exercise Intervention (Type, Duration, Frequency and Intensity)	Main Results
Northey et al. [161], 2019	Pilot RCT	17 BC survivors randomized to HIIT (n = 6), MOD (n = 5), or control (n = 6).	HIIT and MOD groups trained on a cycle ergometer for 3/week for 12 weeks. MOD: 55–65% peak power; HIIT: 105% peak power (90% HRmax) with self-selected active recovery.	All 17 completed follow-up. Adherence was similar (HIIT: 78.7%, MOD: 79.4%). No significant cognitive/cerebrovascular differences, but HIIT showed moderate–large positive effects on memory, executive function, and cerebral blood flow. VO_2_peak ↑ 19.3% (HIIT, d = 1.28); MOD ↑ 5.6% (d = 0.72); control ↓ 2.6%.
Cešeiko et al. [162], 2019	RCT	55 BC patients (stage I–III) randomized to training or control group	Training group performed maximal strength training at 85–90% 1RM, 2×/week for 3 months; control group received standard care without strength training.	Training group ↑ 1RM by 20.4 kg (20%, *p* = 0.001, d = 0.9); control ↓ 8.9 kg (9%, *p* = 0.001, d = 0.5). QoL ↑ 13% in training (*p* = 0.002, d = 0.6), no change in control. Fatigue ↓ 24% in training (*p* = 0.03, d = 0.6); ↑ 25% in control (*p* = 0.02, d = 0.4). Significant between-group differences (*p* ≤ 0.01, d = 0.6–0.9).
Odynets et al. [163], 2019	RCT	115 BC patients were randomly allocated to water exercise (Group A, n = 45), Pilates exercise (Group B, n = 40), and yoga exercise (Group C, n = 30) interventions	All 3 groups attended 144 rehab sessions over 1 year (3×/week). Pilates intensity: 45–60% HRR	QoL improved in all groups. Group A showed higher emotional well-being vs. Group B (+1.40, *p* < 0.05) and Group C (+1.69, *p* < 0.01), and higher BC subscale scores vs. Group B (+2.15, *p* < 0.05). Group C scored higher in social/family well-being vs. Group A (+2.80, *p* < 0.01).
Pasyar et al. [160], 2019	RCT	40 women with BC-related lymphedema were randomized to intervention or control group	Intervention: 8-week yoga program (2×/week instructor-led, 1×/week home practice)	Post-intervention, QoL improved in the yoga group: role functioning (4 weeks, *p* = 0.03), physical and emotional functioning (8 weeks, *p* < 0.05); trends showed increased cognitive, role, physical, and emotional functioning, and reduced fatigue, pain, insomnia, and financial difficulty. No significant change in edema volume.
Scott et al. [164], 2020	RCT	117 BC survivors	Exercise interventions followed linear (LET: 70% VO_2_peak, fixed intensity, 160 min/week) or nonlinear (NLET: 55–95% VO_2_peak, variable intensity, ~120 min/week) dosing, 3–4×/week for 16 weeks, vs. a matched stretching control.	No serious adverse events. Attendance: 64% (control), 75% (LET), 80% (NLET). VO_2_peak ↑ 0.6 ± 1.7 (*p* = 0.05, LET) and 0.8 ± 1.8 (*p* = 0.07, NLET) vs. control. Range: −2.7 to 4.1 (LET), −3.6 to 5.1 (NLET). ~40% were VO_2_peak responders (Δ ≥ 1.32). NLET improved all patient-reported outcomes vs. control
Cešeiko et al. [165], 2020	RCT	55 stage I–III BC patients, scheduled for adjuvant therapy, were randomized to maximal strength training or control.	Maximal strength training group: 4 × 4 leg press at 85–90% 1RM, 2×/week for 12 weeks	Maximal strength training group improved 1RM (+20 ± 8%), walking economy (+9 ± 8%), time to exhaustion (+9 ± 8%), 6MWD (+10 ± 7%), chair rise (+30 ± 20%), and stair climb (+12 ± 7%; all *p* < 0.001); control declined in all. They also maintained quadriceps mass (−7 ± 10% in control, *p* < 0.001). Changes in 1RM strongly correlated with functional gains (r = 0.75–0.81, *p* < 0.001)
Basha et al. [158], 2022	RCT	60 patients with BC-related lymphedema were randomly divided into two groups: the Xbox Kinect group received VR Kinect-based games (n = 30) and resistance exercise group received resistance training (n = 30).	Intervention: 5 sessions/week for 8 weeks	Xbox Kinect group showed greater improvements in pain intensity (VAS), upper limb disability (DASH), shoulder range of motion (*p* < 0.001), bodily pain (*p* = 0.002), general health (*p* < 0.001), and vitality (*p* = 0.006). Resistance exercise group showed greater gains in shoulder flexion (*p* = 0.002), external rotation (*p* = 0.004), abduction, and handgrip strength (*p* < 0.001)
Park et al. [159], 2023	RCT	20 patients with BC-related lymphedema were randomized to progressive resistance exercise group or self-home resistance exercise group	Both groups performed Thera-band resistance exercises. Sessions were 50 min, 3×/week for 6 weeks. Same investigator rated pre/post outcomes.	No significant intergroup difference in edema volume, but significant intragroup change. Both groups showed significant inter- and intragroup improvements in handgrip strength and upper extremity function. QoL improved significantly in progressive resistance exercise group (global health, physical, role, cognitive function, dyspnea); role function and global QoL differed significantly between groups. No QoL changes in self-home resistance exercise group.
Soriano-Maldonado et al. [166,167], 2023	RCT	60 BC survivors were randomized to intervention or control group	Experimental group: 24 resistance training sessions over 12 weeks (2 weeks individual + 10 weeks micro-group) at 50%–65% HRR + 10,000 steps/day. Control group: 10,000 steps/day only.	32 participants in resistance training group (29 ≥ 75% attendance), 28 in control (all completed). Resistance training showed significantly greater gains in full-body strength vs. control (Δ = 0.718; *p* < 0.001, d = 1.04), consistent across upper- (Δ = 0.727) and lower-body (Δ = 0.709) strength. No significant effects on cardiorespiratory fitness, shoulder flexion, fatigue, depression, QoL, or life satisfaction.

Note: RCT: randomized controlled trial; BC: breast cancer; HΙΙΤ: high-intensity interval training; MOD: moderate-intensity continuous training; VO_2_peak: peak of oxygen consumption; QoL: quality of life; HR: heart rate; HRR: Heart Rate Recovery; NLET: nonlinear intensity exercise training; LET: linear intensity exercise training; 6MWD: six-minute walking distance; VAS: Visual Analog Scale; DASH: Disabilities of the Arm, Shoulder, and Hand.

## Data Availability

The data presented in this study are available in the above tables.
